# Hindbrain Effects of L-Glutamate on Gastric Motility in Rats

**DOI:** 10.4021/gr2009.02.1274

**Published:** 2009-01-20

**Authors:** Hong Zhao Sun, Shu Zhen Zhao, Xi Yun Cui, Hong Bin Ai

**Affiliations:** a Key Laboratory of Animal Resistance, College of Life Science, Shandong Normal University, Jinan, Shandong, 250014, China; b Department of Biological Science and Technology, Shandong Institute of Education, Jinan, Shandong, 250013, China

**Keywords:** Dorsal motor nucleus of the vagus, Nucleus of solitary tract, L-Glutamate, Gastric motility

## Abstract

**Background:**

There are no unanimous standpoints about the dorsal motor nucleus of the vagus (DMV) and nucleus of solitary tract (NTS) involving in the regulation of gastric motility up to now.

**Methods:**

In this study, we injected L-Glutamate (L-Glu), an incitant neurotransmitter in the central neural system, into DMV and NTS to further investigate the effects of the two nuclei on gastric motility. A latex balloon connected with a pressure transducer was inserted into the pylorus through the fundus for continuous recording of the change of gastric smooth muscle contractile curves.

**Results:**

L-Glu (10 nmol in 0.1 µl) microinjected into right DMV and NTS significantly inhibited gastric motility. We compared the effects of L-Glu (10 nmol) microinjected into the two nuclei, the L-Glu microinjected into right NTS had the greater inhibitory effect on gastric motility than microinjected into the right DMV. The physiological saline microinjection evoked no significant effect on gastric motility.

**Conclusions:**

L-Glu microinjected into right DMV and NTS evoked significant inhibition on gastric motility in rats. At equal dose of L-Glu, NTS had the greater inhibitory effect than DMV.

## Introduction

It has been reported that dorsal motor nucleus of the vagus (DMV) and nucleus of solitary tract (NTS) can regulate gastric motility. However, whether gastric motility is enhanced or inhibited after the DMV and NTS are excited, the current reports are inconsistent.

Pagani et al. reported that the electrical stimulation (0.1 mA, 0.2 ms, 50 Hz) of the DMV area between 0.56 and 1.56 mm rostral to obex in 20 cats resulted in increases in antral and pyloric contraction, and electrical stimulation of the medial nucleus of the NTS resulted in gastric motility attenuated or no motility responses [1]. Microinjection of the excitatory agent substance P (in 35, 135 or 405 pmol) into the DMV produced a decrease in gastric motility and this decrease was blocked by bilateral vagotomy [2]. Krowicki et al found that excitation of neurons in the DMV rostral to the obex by L-Glutamate (L-GLU) evoked an increase in contractility in rats [3]. Lewis injected 20 nl of 1.0 µM Corticotropin Releasing Factor (CRF) solution into the dorsal vagal complex (DVC, i.e., the DMV and NTS) of the rat and found a very large decrease in gastric motility, evidenced by the decrease of AUC in approximately 83%, which is an indicator of evaluating gastric motility [4]. L-Glu microinjected into the dorsomedial NTS elicited a dose-dependent decrease in tonic gastric pressure and inhibited gastric phasic activity [5]. The inconsistent of these results suggests that further study on the two nuclei modulating gastric motility is needed.

All the two nuclei can regulate gastric motility, however, which one is more important in regulating the is not clear up to now. To clarify these questions, we microinjected L-Glu, a neuron body incitant that can be visualized in hindbrain nuclei, into DMV and NTS respectively to investigate the effects of the two nuclei on gastric motility.

## Materials and Methods

### Animals

Male Wistar rats (260 - 320g) were purchased from Experimental Animal Center of Shandong University. Animals were maintained in a temperature-controlled environment on a 12-h light/dark cycle. They were allowed free access to food and water for one week. Prior to the experiments, animals were fasted for 24 hours, but allowed free access to water. All procedures performed were according to the guidelines of the International Association for the Study of Pain [6].

### Experimental procedures

Animals were anesthetized with an intraperitoneal injection of chloral hydrate (400 mg/kg body weight). Body temperature was maintained at 37 ± 1°C with a radiant heat lamp. A midline laparotomy was performed, and a latex balloon attached to a thin polyethylene tube was inserted into the pylorus through a small incision on the forestomach wall, the polyethylene tube was connected to a pressure transducer. The stomach was inflated by introducing warm physiological saline (PS, 0.5 -1.0 ml) into the balloon to achieve a baseline pressure of 5 - 10 cm H_2_O. Gastric motility curves were recorded by a two-lead physiological recording instrument (LMS-2B, Chengdu Instrument Factory, China).

The animals were then placed in a stereotaxic apparatus (Stoelting 51600, USA), and the dorsal surface of a medulla was exposed by an occipital craniotomy. Glass micropipettes (30 - 50 µm external tip diameter) were prepared from glass capillaries (Dagan, inneapolis, MN), which was vertically inserted into the right DMV and NTS respectively. Stereotaxic coordinates were originally chosen based on histological material presented in Paxinos and Watson [7]. The stereotaxic coordinates of DMV was at a level 13.8 mm posterior to bregma, 0.7 mm right lateral to the midline and a depth of 8.3 mm below the surface of the skull; the stereotaxic of NTS was 13.3 mm, 1.0 mm, 7.9 mm, respectively.

The animals were randomly divided into L-Glu microinjection group and PS microinjection group with 8 rats in each. Firstly, we investigated the effects of L-Glu (10 nmol in 0.1 µl; Sigma Chemical Co) microinjected into right DMV (n = 8) and NTS (n = 8) respectively on gastric motility. For comparison, the effect of equal volume of PS microinjected into the same sites on gastric motor activity was also assessed. Microinjection of drug was performed by pressure and all chemicals were dissolved in PS. The gastric motility was recorded for 30 min before injection, and then L-Glu (0.1 µl) or PS (0.1 µl) was microinjected into the right DMV or NTS continuously within 1 min and followed by recording gastric motility for 30 min.

At the end of the experiments, 2% potamine sky blue (0.1 µl, Sigma Chemical Co) was injected into the same microinjection site. All of the experimental animals were terminated by a bolus intravenous injection of pentobarbital sodium (80 mg/kg). Then, the animals were perfused transcardially with PS and subsequently with 4% paraformaldehyde, the animal brains were removed and fixed in 4% paraformaldehyde with 20% sucrose for at least 2-3 days. Frozen sections of the brain stem (40 µm) were cut and stained with neutral red to determine placement of the micropipette tip in the right DMV and NTS. Photomicrograph images were taken using a microscope (Nikon Optiphot; Nikon) with a digital camera (Magnafire; Optronics, Goleta, CA, USA) attached to a Dell Pentium III Computer. These were then exported into Adobe PhotoShop where they were untouched except for minor adjustments to brightness and contrast.

### Data analysis

The total amplitude, total duration, and motility index of gastric contraction waves within 5 min before microinjection and after microinjection were measured. The motility index was defined as the product of amplitude and duration of every contraction waves. At the same time, inhibitory rate was applied to estimate the changing degree of gastric motility before and after microinjection, namely inhibitory rate = (the value before microinjection – the value after microinjection)/the value before microinjection. All values were analyzed using SPSS11.5 software (SPSS Inc. Chicago, Ill., USA) and presented as mean ± SEM. Statistical analysis was performed by Student’s *t*-test. Significance was accepted at the level of *P* < 0.05.

## Results

### The localization and injection site of the right DMV and NTS

**Figure 1:**
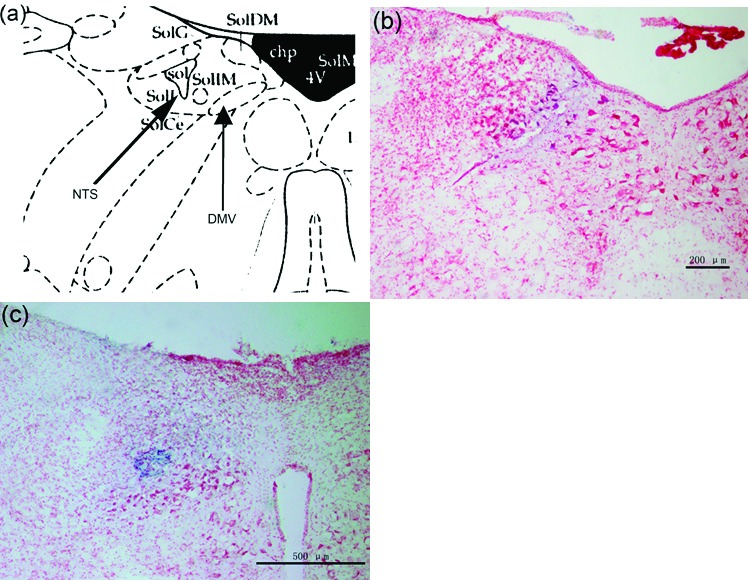
(a) Representative site of DMV and NTS in the brain atlas. (b) Brain stem section stained with neutral red, blue macula indicates DMV. (c) Brain stem section stained with neutral red, blue macula indicates NTS.

The brain stem was stained with neutral red and showed the microinjection site in the right DMV and NTS (Fig. 1).

### L-Glu microinjection into the right DMV

**Figure 2:**
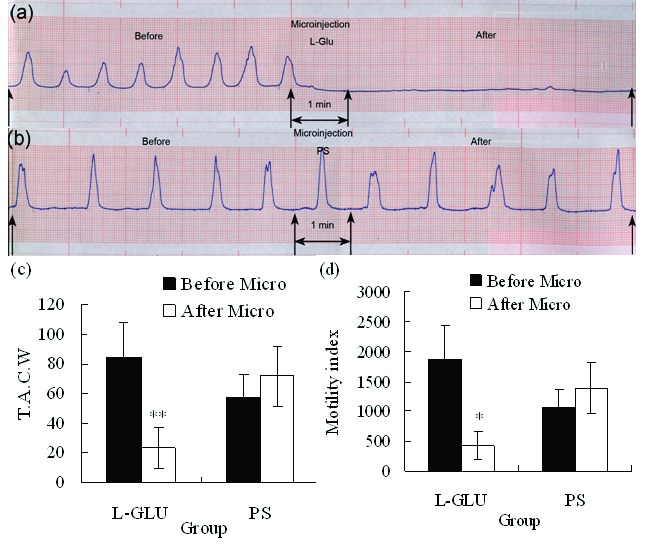
(a) Representative effects of L-Glu microinjected into the right DMV on gastric motility (representing curve from a rat). (b) Representative effects of PS microinjected into the right DMV on gastric motility (representing curve from a rat). (c) TACW before and after microinjection of L-Glu or PS into DMV. (d) Gastric motility index before and after microinjection of L-Glu (or PS into DMV. *P < 0.05; **P < 0.01, versus before microinjection. TACW, total amplitude of contraction waves; Micro, microinjection.

L-Glu microinjected into the rostral of right DMV (rostral to area postrema) evoked significant inhibition on gastric motility (Fig. 2a). Total amplitude of contraction waves decreased from 84.60 ± 23.23 (before microinjection) to 23.20 ± 14.03 mm/5min (*P* < 0.01), gastric motility index decreased from 1866.72 ± 574.66 (before microinjection) to 425.76 ± 231.97 (*P* < 0.05) after L-Glu (10 nmol) was microinjected into right DMV (Fig. 2c, d); however, little changes were noted after PS was injected into the same site (n = 8, Fig. 2b, c, d).

### L-Glu microinjection into the right NTS

**Figure 3:**
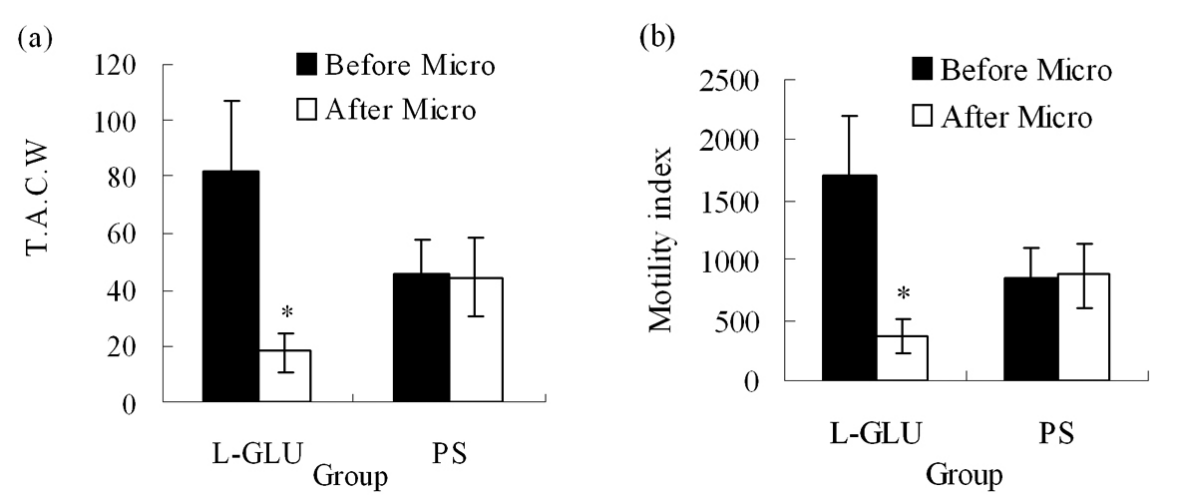
(a) TACW before and after microinjection of L-Glu or PS into NTS. (b) Gastric motility index before and after microinjection of L-Glu or PS into NTS. TACW, total amplitude of contraction waves; Micro, microinjection; *P < 0.05, versus before microinjection.

Microinjection of L-Glu into the medial nucleus of the tractus solitarius (mNTS) also evoked significant inhibition on gastric motility. Total amplitude of contraction waves decreased from 81.58 ± 25.57 (before microinjection) to 18.00 ± 6.83 mm/5min (*P* < 0.05), gastric motility index decreased from 1715.10 ± 484.72 (before microinjection) to 377.00 ± 140.60 (*P* < 0.05) after L-Glu (10 nmol) was microinjected into right NTS (n = 8, Fig. 3a, b); however, little changes were noted after PS was injected into the same site (n=8, Fig. 3a, b).

### Comparison of L-Glu microinjected into the right DMV and NTS

**Figure 4:**
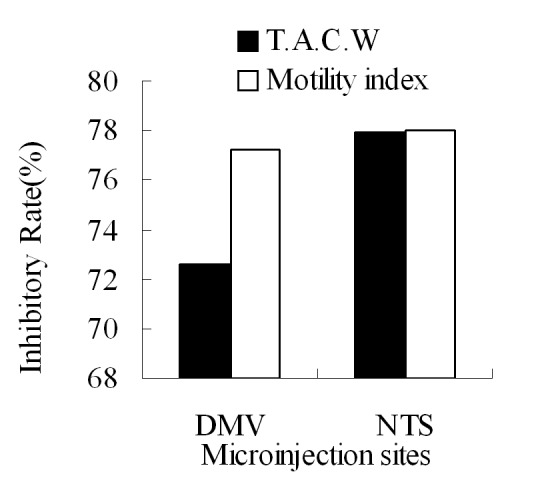
The inhibitory rate of T.A.C.W or gastric motility index after microinjection of L-Glu into the right DMV and NTS respectively. TACW, total amplitude of contraction waves.

By comparison of L-Glu microinjection at equal dose in the right DMV and NTS, NTS had the greater inhibitory effect than DMV on gastric motility (Fig. 4).

## Discussion

Previous study showed that microinjection of L-Glu into DMV altered gastric volume; microinjection into rostral DMV led to gastric contraction, while stimulation of caudal DMV resulted in gastric relaxation in mice [8]. The excitation of neurons in the DMV rostral to the obex by L-Glu evoked an increase in contractility in rats [3]. Cruz reported that in 39 out of 43 rats, microinjection L-Glu into different areas of the DMV, rostral to calamus scriptorius (CS) resulted in vagally-mediated excitatory effects on gastric motility, and microinjection of L-Glu into the DMV caudal to CS produced vagally-mediated inhibition of gastric motility [9]. In this study, L-Glu microinjection into the rostral of right DMV evoked significant inhibition on gastric motility. Our result is consistent with that of Cruz, but different from the others [3, 8]. Cruz et al suggested that the inhibitory effect of L-Glu microinjected into the DMV on gastric motility was likely due to L-Glu diffusing to and exciting NTS neurons from sufficiently high concentrations, they thought that the diffusion to the NTS from the microinjection site in the DMV is likely because these two hindbrain nuclei are so close [9]. We also propose that the inhibitory effect of L-Glu in this study was due to its diffusing to and exciting NTS.

It is well known that the NTS is a viscerosensory nucleus receiving gastroenteric sensory information. Many researchers have reported that the neurons within NTS issue fibers into the DMV, nucleus ambiguous (NA) and constitute synapse connections with the neurons within DMV and NA, thereby modulate their activities, or making the postsynaptic neurons exciting, or inhibiting [10-17].

The L-Glu microinjected into the mNTS produced vagally-mediated inhibition of gastric motility in the rat [9], and the L-Glu microinjected into the dorsomedial NTS elicited a dose-dependent decrease in tonic gastric pressure and inhibited gastric phasic activity [5]. Our findings in this study are consistent with these results. It seems without question that the excitation of NTS can inhibit gastric motility. This inhibitory effect must be via the DMV pathway and NA pathway, the detailed mechanism of this effect is unknown.

Microinjection of L-Glu at equal dose respectively into the right DMV and NTS, NTS had the greater inhibitory effect than DMV had the second effect on gastric motility, these results are conflicting with the traditional opinion that DMV is the main nucleus of modulating gastric motility [18, 19].

According to Cruz’s reports and based on our results, if the inhibitory effect of L-Glu microinjection into the rostral DMV on gastric motility was likely due to L-Glu diffusing to and exciting NTS neurons from sufficiently high concentrations, it implies that the inhibitory effect of the mNTS on gastric motility is likely greater than the excitatory effect of the rostral DMV in the normal physiological state. This is the reason that L-Glu microinjection into the right NTS has the greater inhibitory effect on gastric motility. However, the mechanisms of the two nuclei’s modulation on gastric motility need further more research.
